# Design considerations for development of cuffed endotracheal tube for small airways

**DOI:** 10.1111/pan.15033

**Published:** 2024-11-19

**Authors:** Seamus Maguire, Daniel Wade, James Curley, Sean Morris

**Affiliations:** ^1^ Research and Development Acute Care & Monitoring, Medtronic Athlone Ireland; ^2^ Clinical Research and Medical Science Acute Care & Monitoring, Medtronic Boulder Colorado USA

**Keywords:** cuff position, ETT, high‐volume low‐pressure, pediatric, subglottic, tracheal length

## Abstract

Endotracheal tubes (ETTs) are life‐supporting devices that are designed to maintain a patent airway in patients who are unable to sustain an airway due to illness or injury. Patients with small airways, such as neonates and pediatrics, have unique structural and functional features, making it essential that ETT design considers and executes on these particular needs. Though uncuffed ETTs have historically been preferred for patients younger than eight years of age, advances in cuffed ETT design and construction can be utilized to manufacture ETTs that are optimized for the smallest, most fragile airways. The purpose of this article is to discuss certain design features of cuffed ETTs in respect to small airways.

## 1 INTRODUCTION

Endotracheal tube (ETT) design centers on maintaining a patent airway to support life in patients who are unable to sustain an airway for ventilation. Historically, ETT design decisions have primarily been informed by adult airway anatomy and ventilation needs, with use supported by data collected from adult patients.^1^, ^2^ This, however, does not capture the unique and complex needs of neonatal and pediatric patients with small airways. Compounding these unique needs, premature birth is associated with a plethora of interventions, including surgery and invasive mechanical ventilation. This population is expected to grow in the future with increased rates of live preterm births observed due to improved medical care.^3^ In response to these observations, continual design changes focused on small airways are required to ensure ETTs are appropriately designed with these patients in mind.

Previous reports have described design limitations of ETTs intended for use in the pediatric population, including evaluation of ETT design compared against pediatric airway anatomy.^4^, ^5^, ^6^, ^7^ An analysis of preservative‐free autopsy specimens of the larynx and trachea from 30 Caucasian children highlighted misalignment of traditionally designed ETTs with the airway anatomy of pediatric patients,^8^ concluding that ETTs intended for use in pediatric patients are poorly designed. Further study of the pediatric airway anatomy has continued over the past 20 years and has allowed providers to adopt the use of cuffed ETTs in some of the most vulnerable patients,^9^, ^10^, ^11^ though limitations around the availability of smaller‐sized cuffed ETTs have been noted.^12^ This continued interest in the airways of pediatric patients spurs innovation and design refinements that benefit patient care. Increased awareness of the design requirement for ETTs specifically for use in pediatric care has led the International Organization for Standardization (ISO) to revise the ‘Tracheal tubes and connectors’ standard (ISO 5361) which governs the design requirements for ETTs.^13^ The purpose of this article is to discuss certain design features of cuffed ETTs in respect to small airways.

## 2 ENDOTRACHEAL TUBE DESIGN CONSIDERATIONS FOR PEDIATRIC AIRWAYS

The small airways of infant and pediatric patients have characteristics distinct from the larger airways of adults. The development of ETTs available for use in this patient population requires an understanding of the unique anatomical features and boundaries that constrain ETT design. Though an exhaustive review of pediatric anatomical airway literature is beyond the scope of this paper, we refer the reader to the cited papers for further reading on the topic.^4^, ^5^, ^12^, ^14^, ^15^, ^16^

### 2.1 Endotracheal tube length

The limited length of the trachea in pediatric patients is a key consideration in ETT design. Tracheal length and distance between carina and cricoid cartilage specific to small airways were determined utilizing data from previously published studies and are summarized in Table 1. This data is key for making ETT design choices such as decisions on bevel angle, presence and sizing of Murphy eye, and cuff length and position.

**TABLE 1:** Endotracheal measurements and suggested endotracheal tube sizing based on regression analyses.

Age range (years)	Mean tracheal length^a^ (min – max) (mm)	2/3of mean tracheal length (min – max) (mm)	Mean subglottic length^b^ (mm)	Recommended endotracheal tube size^c^	Dimension ‘C’^d^
Premature				2.5	
<1	45.6 (36.4–52.2)	24.3 (18.2–28.6)	8.8	3.0	24
1–2	50.5 (41.8–60.5)	27.0 (21.3–33.6)	9.5	3.5	27
2–4	58 (49.2–73.9)	31.3 (25.5–41.8)	10.5	4.0	31
4–6	65.2 (49.4–87.6)	35.3 (24.9–50.1)	11.7	4.5	35
6–8	76 (52.3–94.7)	41.4 (25.8–53.7)	13.3	5.0	41
8–10	83.6 (67–98.1)	45.7 (34.7–55.2)	14.4	5.5	46
10–12	83.4 (72.8–105.4)	47.3 (40.3–61.8)	11.7	6.0	48
12–14	90.8 (82.2–116.4)	51.6 (45.9–68.5)	12.7	6.5	52
14–16	103.4 (82.9–116.6)	59.3 (45.7–68.0)	13.6	7.0	59
16–18	123	72.0	13.9	7.5	
18–20	124.5	72.5	14.6	8.0	

^a^ Tracheal length as measured from vocal cord to carina. Tracheal lengths taken from Weiss, Knirsch, Kretschmar, Dullenkopf, Tomaske, Balmer, Stutz, Gerber, and Berger^30^ for ages 0–16 years. Tracheal length taken from Griscom N. T.^53^ for age categories 0–20 years.

^b^ The regression equation 7.8 + 0.03*age (months) was used to calculate subglottic length for all the ages. Subglottic length taken from Sirisopana M^20^ for ages 0–3 years and^52^ for ages 0–5 years.

^c^ Dimension ‘C’ calculated using the data in the analysis above was larger for the 7.5 and 8.0 tracheal tubes. Original dimension ‘C’ for these sizes were retained.

^d^ 3.0–7.0 recommended endotracheal tube sizes taken from Weiss, Knirsch, Kretschmar, Dullenkopf, Tomaske, Balmer, Stutz, Gerber, and Berger.^30^

### 2.2 Cuff position

**FIGURE 1:**
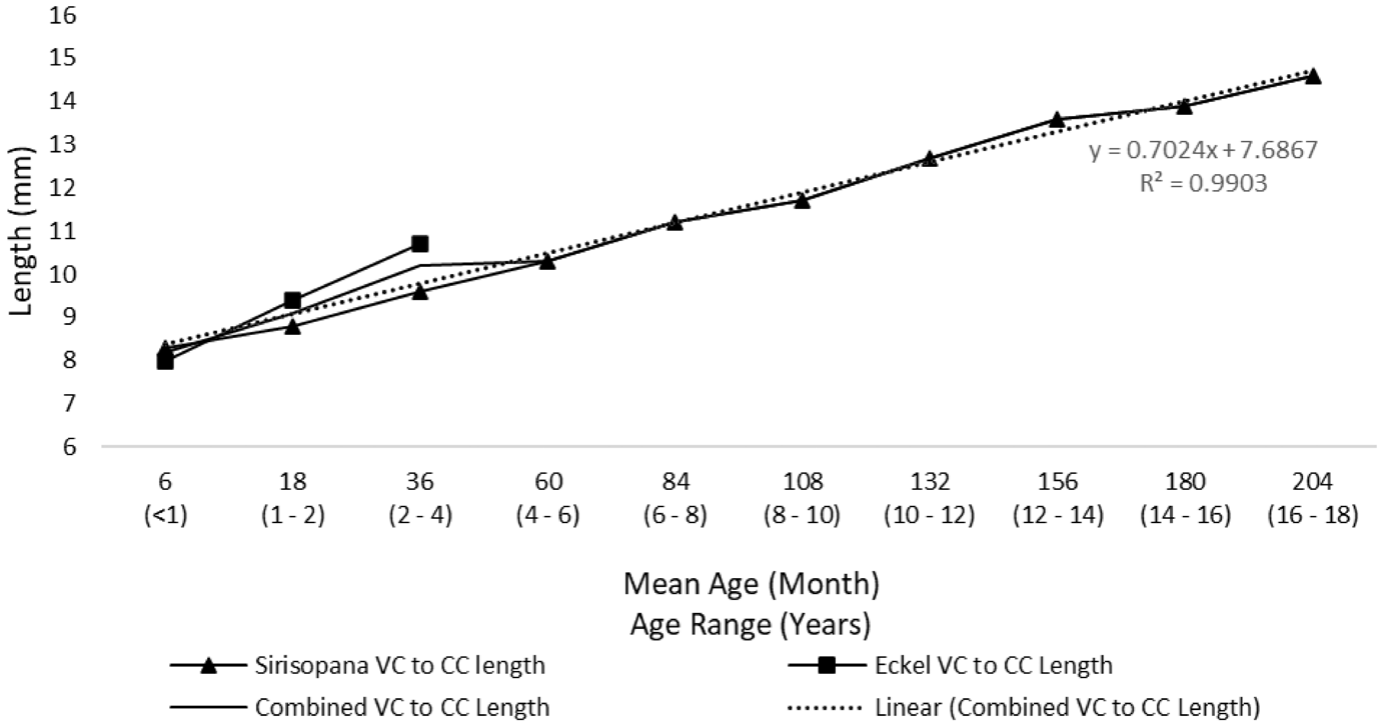
Calculated length of vocal cord to end of cricoid cartilage by age using measurements from previous publications. Using data from Sirisopana et. al.^20^ and Eckel et. al.,^52^ tracheal length from the vocal cords to the end of the cricoid cartilage is plotted across age. This length represents tracheal length that is not appropriate for endotracheal tube placement. The solid black line with triangles represents the tracheal length from vocal cords to the end of the cricoid cartilage as presented by Sirisopana et. al.^20^ The solid black line with squares represents the tracheal length from vocal cords to the end of the cricoid cartilage as presented by Eckel et. al..^52^ The solid black line represents mean tracheal length from vocal cords to the end of the cricoid cartilage for ages 0–36 months, using data from Sirisopana et. al.^20^ and Eckel et. al. ^52^ The dotted black line represents the regression line of tracheal length from vocal cords to the end of the cricoid cartilage to the carina by age. CC, cricoid cartilage; VC, vocal cords.

**FIGURE 2:**
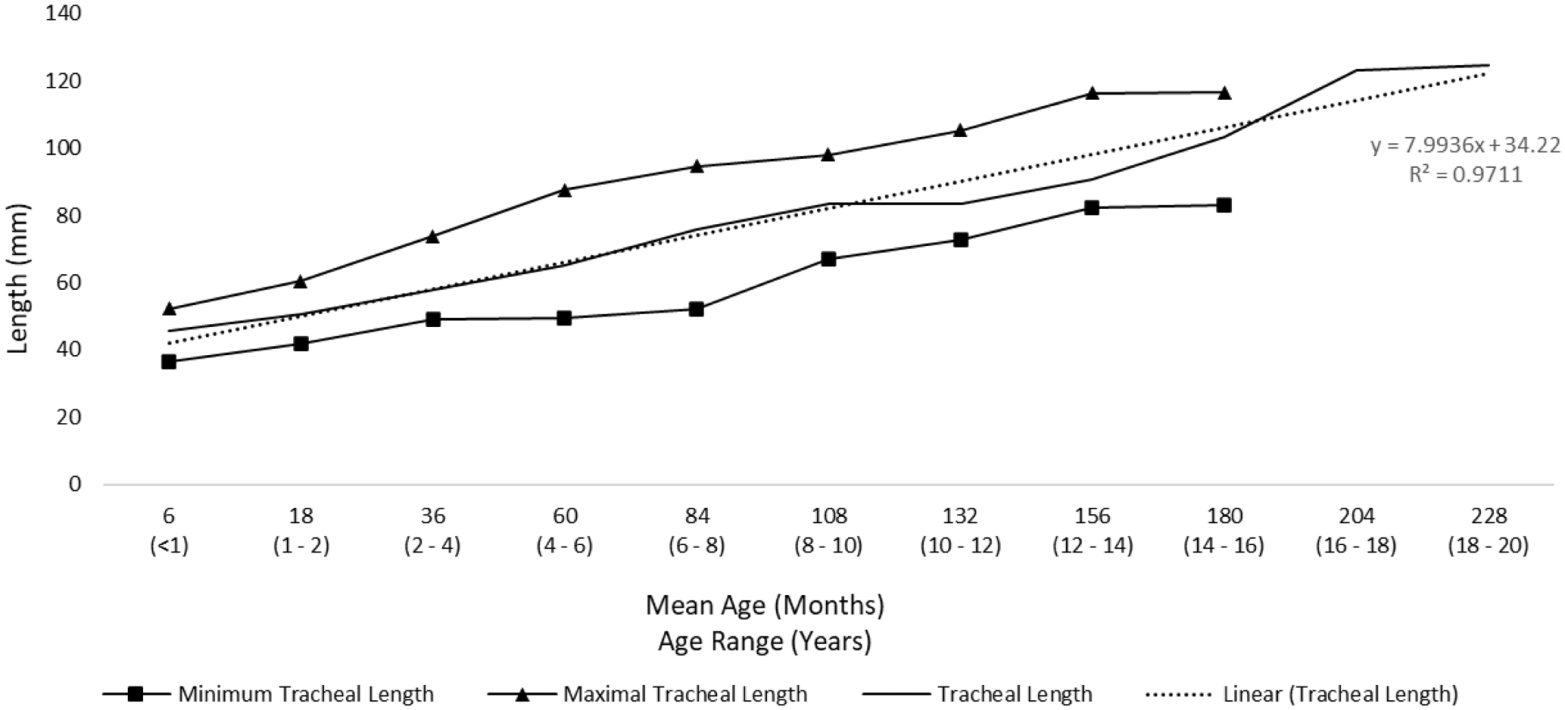
Calculated tracheal lengths by age using measurements from previous publications. Using data from Griscom & Wohl^53^ and Weiss et. al.,^30^ total tracheal length is plotted across age. The solid black line with triangles represents maximal tracheal length by age. The solid black line with squares represents minimum tracheal length by age. The solid black line represents mean tracheal length across age. The dotted black line represents the regression line of tracheal length by age.

**FIGURE 3:**
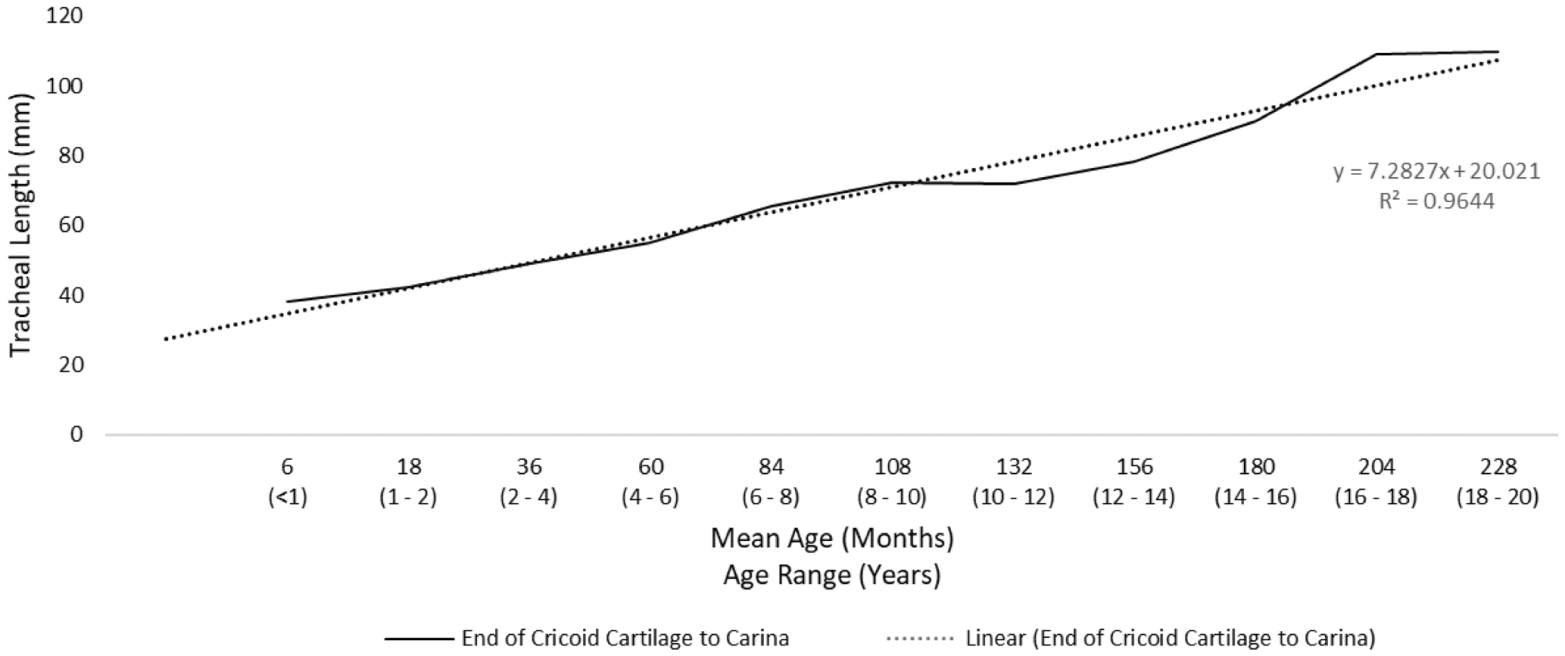
Available tracheal length for cuff inflation from end of cricoid to carina. Tracheal length from the end of cricoid cartilage to the carina was determined by taking total tracheal length and subtracting out the length of the vocal cords and end of the cricoid cartilage, which is considered unusable space for endotracheal tube placement. This measure provides the tracheal length available for endotracheal tube placement. The solid black line represents the tracheal length from the end of the cricoid cartilage to the carina by age. The dotted black line represents the regression line of tracheal length from the end of the cricoid to the carina by age.

With the available length between the cricoid and the carina established, the available tracheal length for cuff inflation was then determined. One of the key shortcomings identified in a number of cuffed ETTs is the position and length of the cuff.^4^ When the tip of the ETT is placed mid‐trachea and the cuff inflated, the inflated portion of the cuff could contact the patient's vocal cords, resulting in an increased risk of injury. Ideally, the cuff should not inflate above the level of the cricoid cartilage.^17^, ^18^ To avoid the possibility of cuff inflation at the cricoid cartilage, we examined the distance from the vocal cords to the end of the cricoid cartilage. As depicted in Figure 1, we utilized anatomical data available from previous studies including infant and pediatric measurements.^19^, ^20^ Data was available for individuals aged 60 months and younger. As no data was available for anatomies above 60 months, the extrapolation of the regression equation was used to predict the length of the vocal cords to the end of the cricoid cartilage (subglottic) for all age categories. Figure 2 depicts the calculated tracheal lengths by age using measurements, and Figure 3 depicts the length available from the end of the cricoid cartilage to the carina.

### 2.3 Murphy eye

**FIGURE 4:**
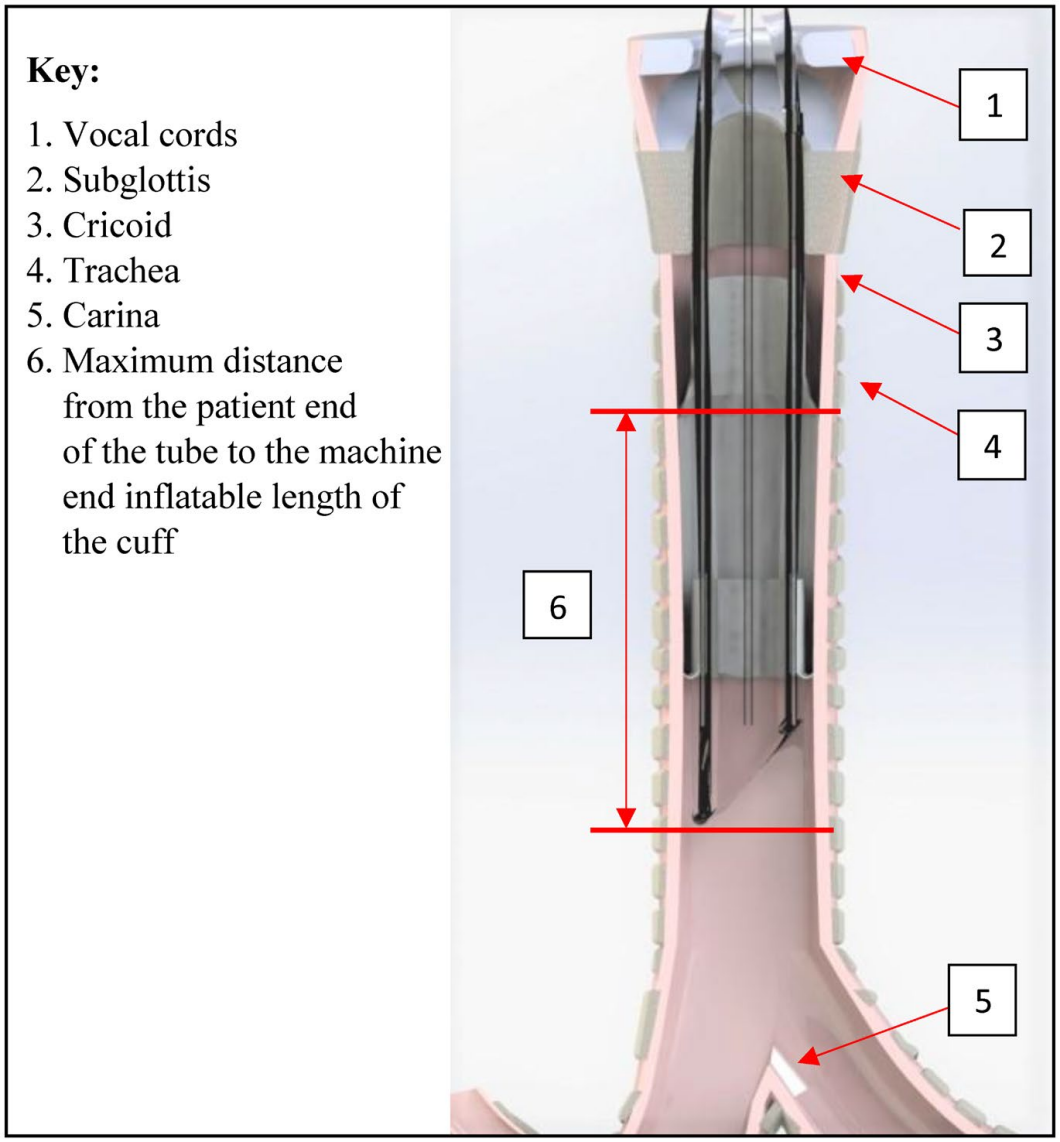
Graphical representation of a Cuffed Endotracheal Tube in the Trachea.

The Murphy eye is a traditional feature of ETTs, meant as a safety feature to provide a secondary vent in the case of complete ETT occlusion.^21^, ^22^ Given adult airway anatomy, the distance between the cricoid and carina allows enough length for the ETT to accommodate an appropriately positioned cuff and Murphy eye. In small airways, the distance between the cricoid and carina is much shorter, resulting in an ETT length that cannot accommodate both an appropriately placed cuff and Murphy eye, as seen in Figure 4. Therefore, design decisions to address this limitation in the available space need to be considered for ETTs meant for small airways.

Though no large observational or randomized control trial were identified by the authors, a few case reports have detailed incidences of ETT occlusion in cases where an ETT lacking a Murphy eye was used, in both adults^23^, ^24^, ^25^, ^26^ and children.^27^, ^28^ ETT occlusion may lead to disrupted gas exchange, reintubation, and complications or adverse events. Though the severity of these events is notable and health care providers should be aware of these potential complications when using an ETT lacking a Murphy eye, they do appear rare. These observations have led some to suggest that ETTs without a Murphy eye may not be appropriate for all use cases, such as longer surgeries.^28^ Cuff inflation at the vocal cords due to poor design and/or improper ETT placement has been associated with tracheal injury, with some evidence to suggest that ETTs with cuffs positioned closer to the tip having greater odds of correct placement.^17^, ^29^ Given all this, it is the opinion of the authors that priority be placed on pushing the cuff more distally at the expense of the Murphy eye.

### 2.4 Depth of insertion

In order to define the dimensional constraints applicable for the design of an ETT, the depth of insertion of the ETT (position within the trachea) needs to be determined. Specifically, the length of ETT that is positioned beyond the vocal cords and unable to be visualized once placed must be considered. If it is assumed that the ETT is inserted into the trachea to the point where the tip of the tube is located at a plane which is 2/3 the total available length between the carina and the cricoid, then the maximum distance from the tip of the ETT to the cricoid can be established. This provides the maximum distance which the inflatable portion of the ETT cuff can be located from the tip of the ETT. This maximum cuff distance is represented by “6” in Figure 4.

It should be noted that the presence of glottic depth markings serve as an indicator to the clinician to assist in locating the ETT tip beyond the vocal cords. If these marks are placed on the shaft of the ETT in a meaningful way, they reflect the anatomical structures of the patients for whom they were designed. Glottic depth marks that are positioned too distally (too close to the tip of the ETT) may result in the tip of the ETT not being inserted far enough beyond the vocal cords. In the case of a cuffed tube, this could result in the cuff being inflated in the narrowest portion of the airway or even partially within the vocal cords. In order to calculate tracheal length and determine where clinically relevant markings should reside on the shaft of the device, the Sirisopana regression equation^20^ (length of subglottic airway (mm) = 7.8 + 0.03*age in months) was used to determine the subglottic airway lengths and break that down into segments as they appear in Figure 4.

With patient movement, particularly head extension, this may result in an increased risk of accidental extubation.^18^, ^30^ Conversely, a glottic depth mark that is too proximal (too far from the tip) can result in the tip of the ETT being inserted too close to the carina. To help guide practitioners in safely positioning the ETT in the trachea, it is vital that the depth markings are based on anatomical data.

### 2.5 Cuff shape

If the decision is made to use a cuffed ETT in small airways, the design of the cuff is an important consideration. When inflated, the cuff conforms to the shape of the trachea providing a means of sealing the trachea to help prevent aspiration, optimize delivery of positive pressure ventilation,^16^ and decrease leakage of inhaled anesthetics to minimize air pollution and anesthetic use.^31^

Traditionally, there have been two categories of cuff type: tight to shaft (TTS) and high volume low pressure (HVLP) cuffs. A TTS cuff is manufactured from a high‐elastic polymer that will expand to fill the airway. The advantage of a TTS cuff is that it will form a seal in the trachea with no folds or creases in the cuff at the junction of the cuff and the tracheal wall. A noted disadvantage is the relatively high intra cuff pressure needed to overcome the tensile forces in the elastic material for it to expand. Therefore, as energy is consumed in stretching the material, there is no correlation between the intra cuff pressure, which can be monitored by the clinician, and the force applied to the tracheal wall. Traditional HVLP cuffs are constructed with a relatively non‐elastic material that in free air has a diameter greater than that of the patient's trachea. The advantage of HVLP cuffs is that the cuff can be inflated to create a seal in the trachea at low pressures. No energy is used to overcome the tensile forces in the cuff material, and therefore there is a direct correlation with the intra cuff pressure and the force applied to the wall of the trachea. It is the ability to infer the force on the tracheal wall that has led to HVLP cuffs being the predominant cuff type in clinical use. The disadvantage of HVLP is that as the diameter of the cuff is larger than the diameter of the patient's trachea folds or creases in the cuff appear at the junction of the cuff and the tracheal wall. These folds or creases have been noted to contribute to micro‐aspiration.^32^, ^33^ Continued advances in the design and manufacturing of HVLP cuffs aims to optimize advantages, while minimizing drawbacks.

**FIGURE 5:**
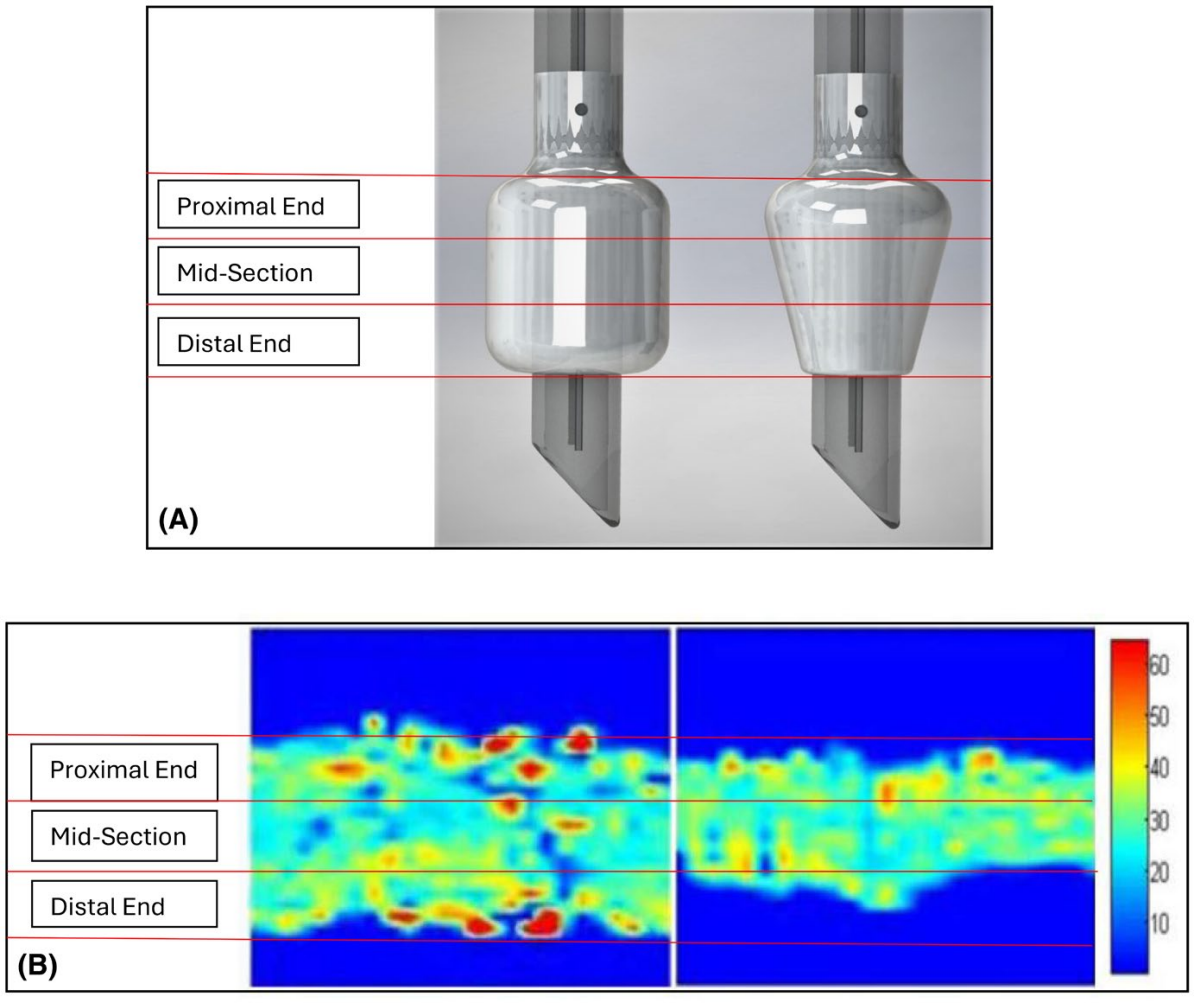
(A) Design features of cylindrical (left) and taper (right) shaped endotracheal cuffs. Pressure profile of barrel shaped cuff (left) and taper shaped cuff (right). (B) The barrel shaped cuff shows a larger contact area with the wall, a larger range of pressures and a higher frequency areas of low pressure and areas of high pressure.^32^

One such optimization is the introduction of cuffs referred to as low volume low pressure cuffs (LPLV). There are two primary shapes of ETT cuffs currently used: cylindrical (barrel‐shaped) HVLP cuffs and tapered (Figure 5) LVLP cuffs. There is evidence to suggest that a tapered cuff design can reduce strain on the tracheal wall compared to a cylindrical cuff design, potentially due to a smaller contact area and more uniform transmitted pressure.^32^, ^34^ A study comparing the traditional HVLP cylindrical cuff to a LVLP tapered cuff using a porcine model found that the tapered cuff shape resulted in less tracheal injury than the cylindrical‐shaped cuff.^32^, ^35^ Even superficial damage to tracheal tissue is of clinical interest as it can increase susceptibility to pathogens, disrupt mucus transport, and be the start of ulceration and fibrosis.^36^, ^37^

### 2.6 Resting cuff diameter

It is proposed by Fischer et al. that the ideal resting cuff diameter for an ETT should be one that is approximately 120% of the largest cross‐sectional area of an age‐related trachea.^15^ The Fischer et al. study reports that there is considerable heterogeneity in resting cuff diameters among different manufactures and that some resting cuff diameters are less than the maximum mid‐tracheal lateral dimeter for the age range in which the ETT size would be selected based on the Motoyama and Khine recommendation for tube size selection. The study also reports that some resting cuff diameters are larger than expected. An inadequate resting cuff diameter may result in hazardous situation related to air leak and the use of excessive intra‐cuff pressures whereas oversized resting cuff diameters may result is risks associated with tracheal injury, difficulty in intubation, and the inability to test for air leak when the cuff is deflated, especially in those with small airways.^38^, ^39^ These findings highlight the need for resting cuff diameter to be informed by pediatric anatomy, along with the importance in the selection of ETT sizes for patient age range. Given the risks associated with oversized resting cuff diameters, smaller cuffed ETT options may prove useful when caring for smaller patients.

### 2.7 Material

ETTs are manufactured from polymers which contain multiple chemicals. During use, these chemicals may be absorbed into the patient. Given the developmental processes and lower body weight present in the neonatal and pediatric populations, the dose to patient needs to be carefully assessed and potentially harmful substances should be removed from the polymers. One such chemical is Di‐2‐ethylhexyl phthalate, known as DEHP. This chemical is used to increase the flexibility of plastics, including those used for ETTs. DEHP is a noted endocrine disturber and considered toxic to humans.^40^ Importantly, children have been shown to absorb, metabolize, and retain DEHP in clinical settings. Therefore, non‐DEHP containing ETT are desirable.

In current practice, the main materials utilized for ETT cuff construction are polyvinyl chloride (PVC) and polyurethane (PU). PU is notably thinner than PVC, allowing for slim cuff wall construction that maintains cuff integrity with less channel formation due to cuff folding.^41^, ^42^ As such, PU cuffs have been shown to better seal the airway compared to PVC, preventing fluid leakage in in vitro trials.^34^, ^41^ Several studies have found an association between PU cuff use and lower rates of micro‐aspiration and ventilator‐induced pneumonia (VAP),^43^, ^44^, ^45^ though others have found no difference in VAP rates between PU and PVC cuff use.^46^, ^47^

Cuff pressure is recommended to be maintained in the narrow range of 20–30 cm H_2_O, as cuff underinflation can contribute to micro‐aspiration and cuff hyperinflation can cause tissue damage to the tracheal wall.^48^, ^49^ Therefore, an accurate measure of cuff pressure is a vital component of effective and safe airway management when using a cuffed ETT. The thin wall of PU cuffs, along with inherent physical and chemical features of PU, contributes to condensation of humidified air inside the cuff, which can interfere with accurate cuff pressure measurements, which may cause unintended damage, especially in those with small airways and those who are intubated for a long time.^42^, ^50^, ^51^ Further, the polymer cost of PU is higher than PVC, and the manufacturing process is more challenging. Therefore, utilizing PU adds notable costs to the final product. This economic consideration may continue to be a barrier to manufacturers and clinicians in utilizing PU cuffed ETTs.

Finally, the configuration of the cuff, when bonded to the ETT shaft, can have a significant impact on the proximity of the cuff to the tip of the ETT. This configuration involves inverting the end of the cuff back into the inflatable portion. This manufacturing technique effectively shortens the overall length of the cuff by up to 4 mm, allowing the cuff to be placed closer to the distal tip of the ETT, allowing for a greater margin of safety in the pediatric trachea.

## 3 CONCLUSION

Improvements in medical care have allowed for more and earlier live preterm births, which continues to expand the needs of the youngest and smallest patients. Design limitations of ETTs intended for use in the pediatric population, including evaluation of ETT design compared against pediatric airway anatomy, have been well described in the literature. New insights into pediatric airway anatomy combined with advancements in manufacturing techniques have spurred ETT design and construction that are optimized for neonate and pediatric patients.

## AUTHOR CONTRIBUTIONS

Seamus Maguire contributed to the design, data analysis and interpretation, writing, and final approval of the manuscript. Daniel Wade contributed to clinical interpretation of data, writing, and final approval of the manuscript. James Curley contributed to the design, data analysis and interpretation, writing, and final approval of the manuscript. Sean Morris contributed to the design, data analysis, and interpretation, reviewing, and final approval of the manuscript.

## FUNDING INFORMATION

All activities presented in this report were funded by and completed at Medtronic. Medtronic manufactures and sells endotracheal tubes, including those specifically designed for small airways, including the Shiley Pediatric Oral Nasal Endotracheal tube with TaperGuard Cuff, Non DEHP. All authors are full‐time employees of Medtronic.

## CONFLICT OF INTEREST STATEMENT

All work completed in this report were sponsored and funded by Medtronic. Medtronic manufactures and sells endotracheal tubes, including those specifically designed for small airways, including the Covidien Shiley Pediatric Oral Nasal Endotracheal tube with TaperGuard Cuff, Non DEHP. All authors are full‐time employees of Medtronic.

## Data Availability

The data that support the findings of this study are available within the manuscript.
